# Exploring the Biological Connection Between Tau and PrP^C^ in Neuronal Cells: GSK3β as a Possible Key Player

**DOI:** 10.1007/s12035-025-05163-2

**Published:** 2025-06-28

**Authors:** Rosalina Gavín, José Antonio del Río

**Affiliations:** 1 Department of Cell Biology, Physiology and Immunology, Faculty of Biology, University of Barcelona, Avinguda Diagonal 643, 08028 Barcelona, Spain; 2 Molecular and Cellular Neurobiotechnology, Institute for Bioengineering of Catalonia, Baldiri and Reixac, 15-21, 08028 Barcelona, Spain; 3 Institute of Neuroscience, University of Barcelona, Barcelona, Spain; 4 Center for Networked Biomedical Research in Neurodegenerative Diseases (CIBERNED), Barcelona-Madrid, Spain

**Keywords:** Cellular prion protein, Tau, Neurodegeneration, Neurogenesis

## Abstract

Cellular prion protein (PrP^C^) and tau are highly expressed in the brain and overlap at the cellular level in neurons. Both proteins contribute directly to neurodegeneration processes in a misfolding state, although in their natural conformation, they play important roles in neurogenesis that could have a common link according to the recent literature. In this sense, it is well known that the proteinase-K resistant PrP^C^ isoform (PrP^Sc^), the prion, is the causal agent of prionopathies. And misfolded tau, which is responsible for tauopathies, is considered “prion-like” because it displays similar behavior to prions in terms of self-aggregation and spreading properties. At the physiological level, PrP^C^ potentiates neuronal differentiation while tau intervenes in axonal maturation and elongation. Likewise, recent studies from our laboratory reported that PrP^C^ directly affects the alternative splicing of tau through inhibition of GSK3β while tau, in turn, can regulate *PRNP* transcription. In this review, we first describe the biology and physiological roles of PrP^C^ and tau in the central nervous system (CNS). Second, in the effort to improve our understanding of a possible cooperation between them in various cellular circumstances, we also discuss the molecular convergence points between PrP^C^ and tau in neurodegeneration and in natural neuronal physiology.

**Supplementary Information:**

The online version contains supplementary material available at 10.1007/s12035-025-05163-2.

## Cellular Prion Protein (PrP^C^)

### Location, Expression, and Gene

Cellular prion protein (PrP^C^) is a cell surface glycoprotein linked to the plasma membrane by a glycosylphosphatidylinositol (GPI) anchor. PrP^C^ is expressed in a variety of tissues in mammals, although it is mainly expressed by neurons and glial cells in the adult central nervous system (CNS) and other tissues such as lymphoid tissue and kidney [1–7].

PrP^C^ is encoded by a single-copy gene (*Prnp*) and presents a high level of conservation between species [8], especially in several transcription factor-binding sites of the regulatory region promoter [9]. In addition, in most species, including humans, the gene sequence comprises 2 exons, while cattle, mice, and sheep present an additional exon 3 [8, 10]. However, the coding sequence (CDS) is always in exon 2, and the 5′ untranslated region (UTR) upstream of exon 1, which is quickly spliced, comprising a CpG island and containing the promoter region [11, 12]. In this sense, all characterized *Prnp* promoters appear to be typical housekeeping genes, lacking a TATA box and including Sp1 binding sites [13]. Although two promoter regions have been described in murine neuronal *Prnp* [11], consensus sequences for AP1, AP2, and HSE have also been described upstream from exon 1 in both murine and human cells among other species. In addition, Mahal and colleagues suggest the AP1 binding site as being responsible for higher expression levels in human neurons [14].

### A Brief Examination of Functions of PrP^C^ in the CNS

The location of PrP^C^, its intracellular processing, and its membrane behavior promote its binding to multiple interactors, making it difficult to determine all physiological functions of the protein and its interactions, which remain under study [15, 16]. However, some PrP^C^ functions in brain include several roles in neuroprotection, reviewed in [17]. Briefly, PrP^C^ presents antioxidant activities through copper interaction [18] and modulation of endogenous superoxide dismutase (SOD) [19] and glutathione reductase (GR) activities [20]. In addition, PrP^C^ protects against excitotoxicity through modulation of glutamate receptors [21–24]. Furthermore, an increasing number of studies suggest that PrP^C^ is involved in neuronal differentiation of neural progenitors from different stem cell populations [25–29], a process strongly influenced by glycogen synthase kinase 3β (GSK3β kinase) activity [30]. In this sense, PrP^C^ promotes phosphorylation of GSK3β on serine 9, residue triggering an in vivo reduction of GSK3β activity [31].

In fact, human *PRNP* transcription is decreased during normal aging [32] and in some neuropathological contexts where GSK3β activity is upregulated, such as advanced Alzheimer’s disease (AD) [33, 34].

## Tau Protein

### Expression, Location, and Gene: Alternative Splicing

Microtubule-associated protein tau (MAPT) is a neuronal intracellular protein with a high level of conservation in several species such as mouse, human, bovine, and rat [35, 36]. Human tau is encoded by the *MAPT* gene, which comprises 16 exons on chromosome 17q21 and generates six possible isoforms under a complex alternative splicing [37, 38]. These isoforms are distinguishable by the exclusion or inclusion of a repeat region of exon 10 that generates four (4R) or three (3R) tau microtubule-binding repeats, and both with either no (0N), one (1N), or two (2N) amino-terminal inserts [37, 39, 40]. During embryonic development, specific neuronal types 3R tau are predominant, while in adult neurons, 3R and 4R tau isoforms are present to a similar degree and localized mainly in axons [39, 41]. Several factors that include RNA binding proteins, kinases and miRNAs are involved in the complex control of exon 10 splicing and tau metabolism (see [42] for review). Among these, the GSK3β kinase, which also phosphorylates tau in healthy brain (see below), represents one of these factors [43].

### Physiological Function of Tau

Soluble tau promotes microtubule (MT) stabilization and assembly, interacting dynamically with the cytoskeleton through its MT-binding domain, affecting axonal transport and synaptic plasticity. The regulatory control of tau-MT interaction depends mainly on several kinases and phosphatases [44] since increasing phosphorylation of tau inhibits its binding to MT in a controlled physiological way [45]. Among these kinases, GSK3β, cyclin-dependent kinase 5 (CDK5), and mitogen-activated protein kinases (MAPKs) play significant roles. In addition, when dysregulated, the same kinases also contribute to tau hyperphosphorylation [46, 47]. In contrast, phosphatases play a crucial role in dephosphorylating tau protein, thereby counteracting their hyperphosphorylation. The primary phosphatases involved in tau dephosphorylation are protein phosphatase 2A (PP2A), protein phosphatase 1 (PP1), protein phosphatase 2B (PP2B, also known as calcineurin), and protein phosphatase 5 (PP5) (e.g., [48–50]).

In addition, the different isoforms of tau also play a role in its ability to bind MTs. In this way, 4R tau isoforms have been implicated in neuronal maturation [51], conferring a high level of MT binding to the tau protein [52]. In fact, 4R tau are the most abundant isoforms in fully differentiated adult neurons, presenting reduced plasticity and lower tau phosphorylation levels. In contrast, 3R tau isoforms, which present lower affinity for MT and higher susceptibility to phosphorylation, are increased during periods of prolific synaptogenesis [51].

While MT-stabilization is essential for proper axonal transport, both the local concentration of tau and the balance of the different isoforms have a direct influence on the function of the dynein and kinesin microtubular motors. In this sense, the presence of high tau levels inhibits the binding of motors to MT, although the 3R tau isoform is the more relevant inhibitor [53, 54].

In addition, GSK3β can also directly alter bidirectional axonal transport in mammalian neurons through phosphorylation in both dynein and kinesin motors [55].

## Understanding the Significance of Reviewing PrP^C^ and Tau Proteins

**Fig. 1:**
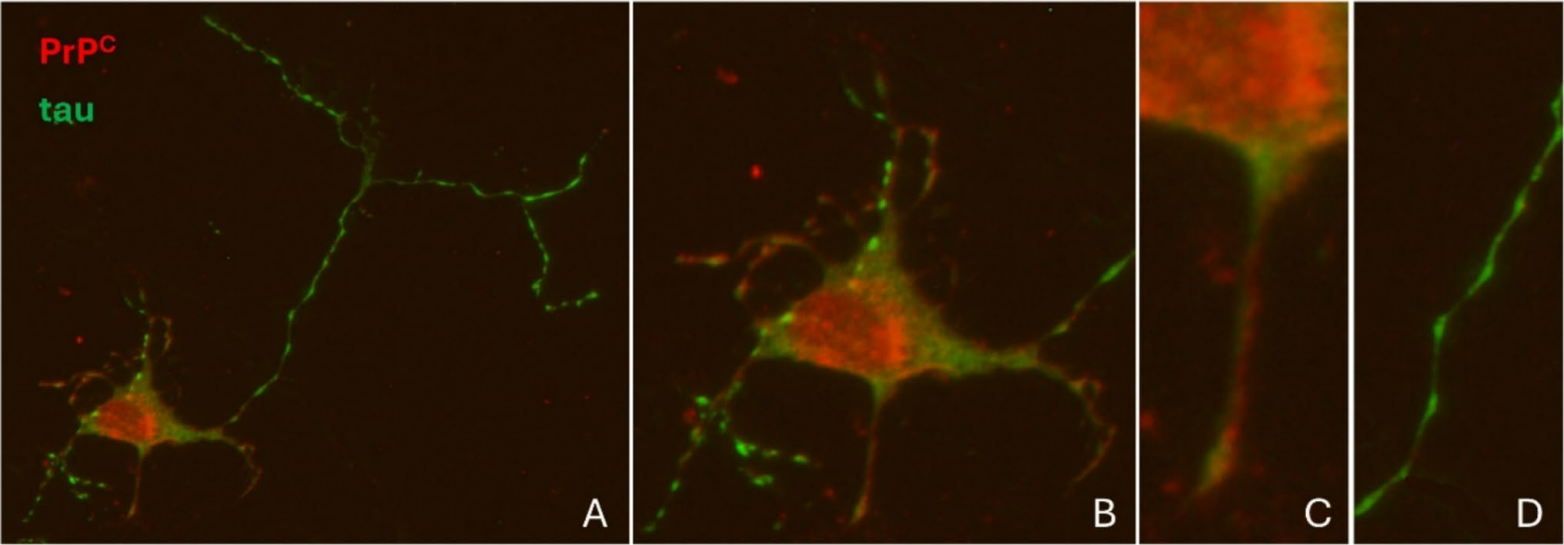
Colocalization of PrP^C^ and tau in neurons. Immunocytochemical detection of PrP^C^ (red fluorescence, 6H4 antibody) and tau (green fluorescence, TAU5 antibody) expression from primary cultures of mouse cortical neurons at 7*DIV*. As widely reported, tau becomes enriched in axons when neurons are developmentally polarized. However, remaining tau expression in neurites and cell body colocalizes with PrP^C^. (**B**)–(**D**) High magnification images of detailed areas shown in **A**; (**B**) soma, (**C**) dendrites, and (**D**) axon

As mentioned above, PrP^C^ and tau are highly expressed in the brain, colocalizing at the cellular level in neurons ([56] and Fig. 1) and in glial cells.

Also, additional contexts suggest a common link between the two proteins at the functional level.

Thus, misfolding of either protein leads to devastating neurodegenerative pathologies that often have points of convergence. In this sense, it is well known the proteinase-K resistant PrP^C^ isoform (PrP^Sc^) is the causal agent of prionopathies [57]. In fact, PrP^Sc^ gave rise to the term prion. Also, misfolded tau, which is responsible for tauopathies, presents similar behavior to prions in terms of self-aggregation and spreading properties [58–62]. It is therefore considered a “prion-like” protein.

In addition, at the physiological level, both play important roles in neurogenesis. In this line, recent studies from our laboratory reported that PrP^C^ directly affects the alternative splicing of tau through inhibition of GSK3β [63] while tau, in turn, can regulate *PRNP* transcription [64].

Clearly, function and dysfunction of PrP^C^ and tau must be analyzed together to uncover a possible cooperation between them in various cellular circumstances.

### Convergence Points Between PrP^C^ and Tau in Neurodegeneration

Briefly, prionopathies, also termed transmissible spongiform encephalopathies (TSEs), are a group of fatal neurodegenerative diseases that comprise genetic, infectious, and sporadic disorders. The well-described TSEs that occur in humans are Kuru, Creutzfeldt-Jakob disease (CJD), Gertsmann-Straussler-Scheinker syndrome (GSS), and fatal familial insomnia (FFI), while other prionopathies have been described in cattle, sheep, and goats, among others [65–68].

On the other hand, tauopathies comprise a heterogeneous group of neuronal or glial pathologies whose common denominator is the presence of pathological aggregates of hyperphosphorylated tau [69, 70]. These aggregates cause increased MT instability, impaired axonal transport, and deficits in synaptic function in neurons [36, 71]. Some of well-described tauopathies include corticobasal degeneration (CBD), globular glial tauopathy (GGT), Pick’s disease (PiD), and Alzheimer’s disease (AD), among others [69, 70]. Additionally, brains affected by these diseases show an altered 3R/4R tau balance [72, 73]. For example: AD (3R + 4R tauopathy); PiD, a prototype 3R tauopathy; and GGT, a 4R tauopathy with characteristic globular and glial tau inclusions. In this sense, GSK3β may be one of the factors implicated in dysregulation of exon 10 alternative splicing, and in any case, it is known that GSK3β is one of the main kinases implicated in the pathological phosphorylation of some of the tau epitopes associated with tauopathies, including AD [74].

In addition to the self-aggregation and spreading properties of PrP^Sc^ and phospho-tau, quite a few studies report the coexistence of the two toxic species in the disease. Moreover, the relevant role of PrP^C^ during various tauopathies has been described.

#### How Does Tau Behave in Prionopathies? Hyperphosphorylated in Parenchyma and Biofluids

Hyperphosphorylated tau has been observed surrounding or colocalizing with PrP^Sc^ plaques in both human cases and animal models. Specifically, tau hyperphosphorylation has been reported in mice infected with bovine spongiform encephalopathy (BSE) and variant Creutzfeldt-Jakob disease (vCJD) [75, 76]. More notably, several studies have identified phospho-tau deposits in patients with Gerstmann-Sträussler-Scheinker syndrome (GSS) [77–80], as well as in sporadic and variant CJD cases [75, 81–83]. Phosphorylated tau has also been detected in biofluids such as cerebrospinal fluid (CSF) and plasma from CJD patients [84, 85].

At the molecular level, dysfunction in the PI3K-Akt-GSK3 signaling pathway is a consistent feature in both in vitro and in vivo models of prion disease [86], and this disruption directly affects tau phosphorylation. In line with this, our group and others have shown GSK3 activation in cultured cells treated with PrP_106–126_, a peptide commonly used to model prion-induced neurotoxicity [87, 88]. These studies demonstrated increased phosphorylation of tau at Ser^396/404^.

Wang et al. [89] further suggested a role for GSK3β in prion pathogenesis by showing its dysregulation in scrapie-infected hamster brains. In this model, GSK3β expression and transcription were reduced, accompanied by lower levels of phospho-tau at Ser^396/404^, despite increased transcription of two tau isoforms.

Recent in vitro evidence also supports a non-amyloid interaction between PrP^Sc^ fibrils and tau monomers [90]. Based on these findings, we propose that the spatial proximity of tau to PrP^Sc^, combined with impaired GSK3β inhibition due to loss of PrP^C^ function, may contribute to tauopathy in certain prion diseases [90].

#### How Does PrP^C^ Behave in Tauopathies? Membrane Receptor for Toxicity with Neuroprotection at Intracellular Level

Among the many known membrane interactions of PrP^C^, it is not surprising that it has been described as a receptor for different forms of extracellular tau (reviewed in [91, 92]). In this sense, soluble aggregated tau species have been reported to bind to PrP^C^ and to inhibit hippocampal long-term potentiation (LTP) in vivo [93] in the same way as described for β-amyloid-derived diffusible ligands (ADDLs) [94]. However, here, we will not discuss the hypothetical toxicity of the phospho-tau-PrP^C^ complex or ADDLs-PrP^C^ association through LTP interference, both of which have been widely debated (reviewed in [17, 95, 96]). We will instead focus on analyzing other biological explanations and consequences of this protein association.

Various in vitro studies have demonstrated the binding of PrP^C^ to various recombinant human tau fibrils containing the four microtubule-binding repeats: full-length tau 441 [97], K18 fragment (amino acids 244–372), and the extended 244–378 fragment [98] [99] or full-length phospho-tau [100]. Importantly, the studies performed in the Legname lab reveal that due to the well-described endocytic cycle of PrP^C^ (e.g., [101, 102]), PrP^C^ is an endocytic receptor of tau aggregates able in turn to feed back into the membrane, resulting in enhanced tau internalization and spreading. But in parallel, they report the capacity of K18 to increase the α-cleavage of PrP^C^ into the neuroprotective fragments N1 (PrP N-terminal: aa 23–110) and C1 (PrP C-terminal: aa 111–231) [98, 99]. They also suggest that N1 could be involved in the reduction of PrP^Sc^ in models of prion infection. In fact, the role of N1 in the reduction of β-amyloid fibrillization and toxicity is already known [103]. It has recently been reported that there is a neuroprotective action of N1 on tau oligomer cytotoxicity through a direct interaction between the soluble fragment of PrP^C^ and oligomers of phosphorylated K18Δ280 [104]. In line with the abovementioned PrP^C^ feedback into the membrane triggered by tau, RNA-seq results from a triple transgenic AD model mouse MAPTnull + APP/PS1 + rTg21221 reveal an increase in PrP^C^ mRNA [105]. Interestingly, PrP^C^ mRNA levels were recovered with tau suppression, suggesting a new association between tau and the physiology of PrP^C^ [105].

In addition to the above-described membrane interaction between tau and PrP^C^, we and others have shown the role of PrP^C^ in downregulating tau levels [33, 106]. In this sense, we demonstrated that PrP^C^ is a relevant factor in controlling tau levels after ADDL treatment and in amyloid burden progression in an APP/PS1 animal model and in human AD-affected brains. Although we were unable to uncover the regulatory mechanism, we ruled out transcriptional control of *MAPT* by PrP^C^, suggesting a post-transcriptional mechanism [33]. The second study mentioned [106] demonstrated, using a cell culture experiment, that PrPC reduces the levels of both 3PO-tau (a mutant form of full-length tau with higher aggregation capabilities) and phospho-tau. The authors suggested that PrP^C^ might regulate the degradation process of 3PO-tau or phospho-tau as a mechanism for attenuating tau production and the consequent toxicity [106].

### Relationship Between PrP^C^ and Tau in Neuronal Physiology

As we have just noted, PrP^C^ and tau present a clear association in a pathological scenario that can help us to better understand the interaction between the two proteins in a healthy neuron.

**Fig. 2:**
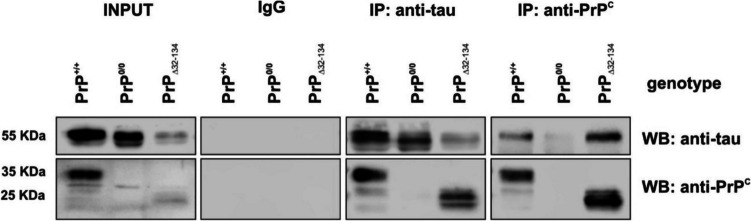
Interaction between native PrP^C^ and tau in mouse brain extracts. Western blot analysis of protein co-immunoprecipitation of PrP^C^ and tau in WT (PrP^+/+^), PrP^0/0^, and PrP^C^ truncated in residues 32–134 (PrP^Δ32−134^) mice. Brain protein extracts from each mouse were immunoprecipitated using anti-tau antibody (TAU5), anti-PrP^C^ antibody (6H4), and IgG as a negative control. After this, the same antibodies were used to blot the co-precipitating protein as well as the precipitating protein. TrueBlot.® anti-mouse IgG HRP secondary antibody was used to avoid detection of heavy and light chains of the immunoprecipitating antibody. Cell extracts before immunoprecipitation (INPUT) are shown in the left panel. Ponceau staining of replicate membranes was used as a loading control (see Supplementary Fig. 1)

In fact, far from a demonstrated colocalization and interaction in disease, tau has been proposed as an intracellular partner of PrP^C^. The complex formed between the non-pathogenic forms of the two proteins has been reported in several studies [106–108]. In this sense, Xiaoping’s lab demonstrated the direct interaction between the two native proteins in brain hamster homogenates with immunoprecipitation analysis [107]. In addition, they used recombinant glutathione S-transferase (GST)-human tau and various fragments of hamster PrP^C^ to develop a pull-down assay. Then, they pointed to residues 23 to 91, which contain the proline/glycin rich octarepeat region (residues 51 to 91), as being responsible for the interaction. Later on, they and others demonstrated interaction between full-length human tau and human PrP^C^, again using recombinant proteins [106, 108]. Moreover, the Xiaoping research group developed an ELISA test to determine that octapeptide repeats (OR) of PrP^C^ directly affected the binding. However, unpublished results from our group on mouse brain extracts, while confirming the interaction between the two native proteins in mouse, rule out the involvement of OR, as the use of an anti-tau antibody to immunoprecipitate PrP^C^ also precipitated the PrP^C^ deleted form into region 32–134 (see Fig. 2).

Although these findings require further validation, they suggest that residues 23–32 (23–30 identical in the mouse and human sequences) are primarily responsible for the interaction between PrP^C^ and tau.

#### Gene Regulatory Control Between the Two Proteins

**Fig. 3:**
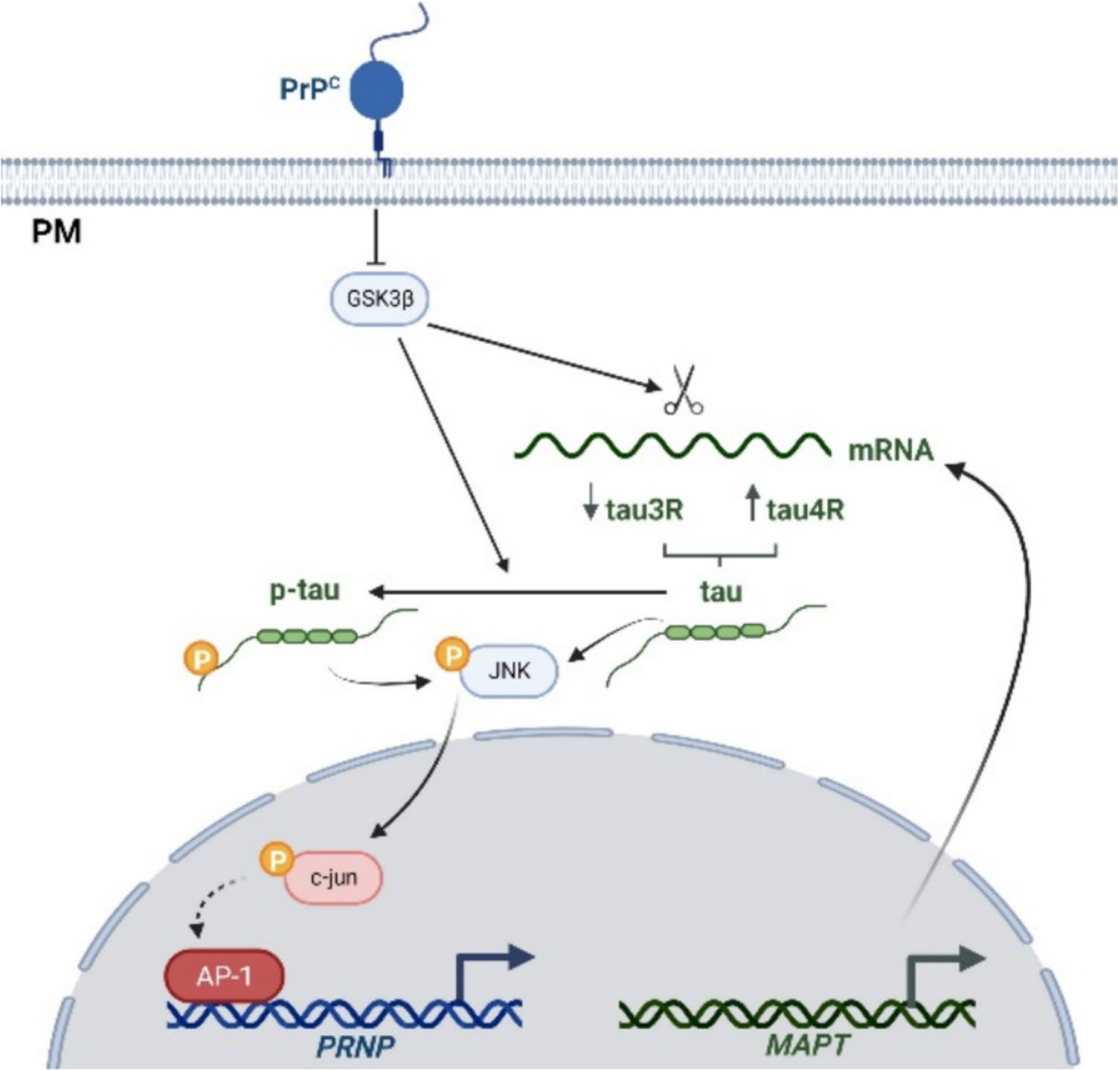
Genetic relationship between PrP^C^ and tau. Schematic representation of the intracellular convergence of PrP^C^ and tau in the transcriptional control of the *PRNP* gene as well as the post-transcriptional control of tau mRNA with GSK3β as a link. Created with BioRender.com

Several transcription factors are involved in the upregulation of the *PRNP* gene by interaction with previously described transcription factor binding sites in its promoter region. Thus, Sp1, MTF-1, and p53 transcription factors mediated the increased *PRNP* activity under oxidative stress conditions with copper as the main actor in triggering increased reactive oxygen species (ROS) [109–111]. In fact, Sp1 can act as a single upregulator of *PRNP* transcriptional levels under hypoxia or copper overload [112]. We reported recently that overexpression or uptake of non-fibrillary variants of tau resulted also in enhanced *PRNP* activity in reporter cells. And in this case, AP-1 was determined to be the main binding site mediating tau effects in parallel to non-specific factors such as Sp1, activated in these circumstances independently of ROS [64]. As noted, the described study was focused on examining the PrP^C^ overexpression found in the early stages of AD, previously described by us and others [33, 113, 114]. Therefore, we developed in vitro experiments to analyze *PRNP* promoter activity under tau transfection as well as extracellular treatments with different recombinant tau forms. Our results indicated that only intracellular tau, either by overexpression or by internalization, was able to increase the activity of the *PRNP* promoter [64]. This is not the case with pathogenic forms of tau, which as we have seen above bind to PrP^C^ extracellularly. Therefore, despite our focus on AD, this could be a physiological response to any situation of intracellular increase in tau transcription, especially in light of the recently described PrP^C^ involvement in the post-transcriptional control of tau by alternative splicing through inhibition of GSK3β [63]. Thus, there must be cellular homeostasis between the two proteins for proper neuron biology (see Fig. 3). In addition, although membrane PrP^C^ may affect the internalization and pathological consequences of extracytosolic toxic tau species, tau, once internalized, may overexpress PrP^C^, increasing its neuroprotective potential.

#### Cooperative Work of Tau and PrP^C^ in Neuronal Differentiation and Axonal Transport

As mentioned above, both PrP^C^ and tau are involved in neurogenic processes. Specifically, PrP^C^ takes part in proliferation, differentiation/maturation, and axon targeting [115], while tau also intervenes in axonal maturation and elongation. In fact, inhibition of GSK3β by PrP^C^ generates an increase in the 4R tau isoforms implicated mainly in neuron maturation [63].

Several studies have revealed increased PrP^C^ levels during ES cell differentiation and brain development in mouse, rat, and cow [26, 116–118]. In addition, some authors have reported that *Prnp* gene expression in the nervous system begins in post-mitotic neural cells that have undergone neuronal differentiation. Interestingly, tau mRNA expression also increases with neuronal maturation, showing low amounts of mRNA and protein even in very early neuronal precursors [119].

On the other hand, all those tau factors that affect its binding on microtubules (differential splicing and phosphorylation) are regulated by GSK3β and, therefore, although only in part, by PrP^C^. This would lead to lower phosphorylation of tau as well as an increase in 4R tau isoforms, thereby affecting the tau binding to MT and consequently the transport capacity. In this sense, preliminary studies by our group point to a decreased average speed per track in mice lacking PrP^C^ after analyzing mitochondrial axonal transport with Mitotracker. And it should be borne in mind that PrP^C^ may cooperate in transport, since its role in inhibiting GSK3β helps to stabilize MTs by reducing tau phosphorylation and increasing 4R tau isoforms, which have a greater affinity for MTs [120]. This also prevents the inhibitory effect of GSK3β on the motor proteins kinesin and dynein [55].

Although the current evidence suggests a cooperative role for PrP^C^ and tau in neuronal differentiation and axonal transport, further studies including functional assays would be instrumental in establishing a causal relationship.

## Concluding Remarks

In this review, we aim to highlight the cooperative interaction between PrP^C^ and tau within the physiological context of neurons. We propose that GSK3β is a key molecular link between the two proteins. Both PrP^C^ and tau have been individually associated with neurodegenerative processes, and so identifying GSK3β as a shared regulatory element offers a novel perspective that can help integrate diverse pathogenic mechanisms. As a result, this review opens new avenues for understanding how changes in one protein may influence the other through this molecular axis. This interplay deserves further investigation, as it could uncover previously unrecognized mechanisms of neurodegeneration and identify new therapeutic targets that target the shared regulatory network rather than focusing solely on each protein individually.

## Supplementary Information

Below is the link to the electronic supplementary material.

Supplementary file1 (JPG 57 KB)

Supplementary file2 (JPG 120 KB)

## Data Availability

No datasets were generated or analysed during the current study.
