# Supplementary figures and images for: Exploring the Biological Connection Between Tau and PrPC in Neuronal Cells: GSK3β as a Possible Key Player
Source: Mol Neurobiol. 2025 Jun 28;62(12):15284–94. doi: 10.1007/s12035-025-05163-2 (PMC12559108; doi:10.1007/s12035-025-05163-2)

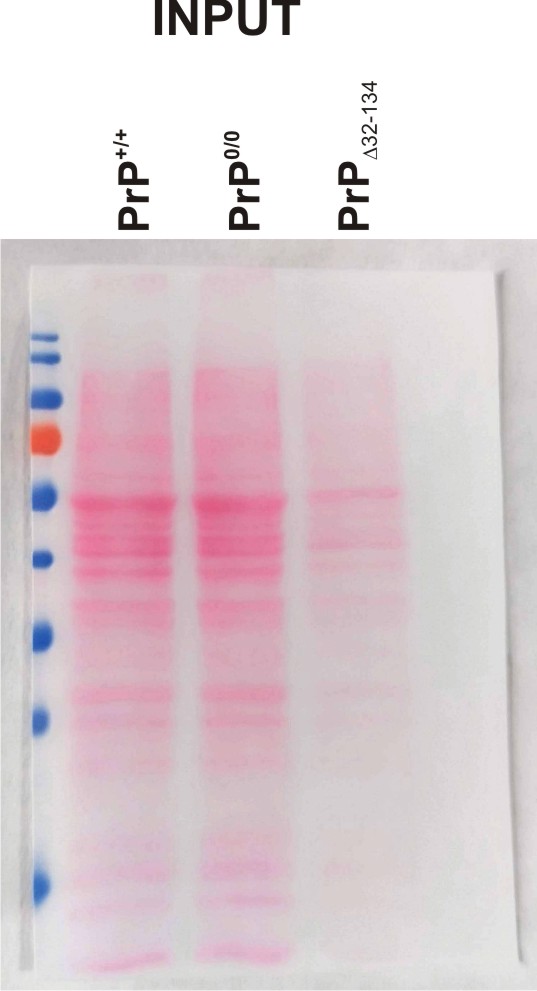

Supplement: Supplementary file 1 — Supplementary file1 (JPG 57 KB) [file 12035_2025_5163_MOESM1_ESM.jpg]

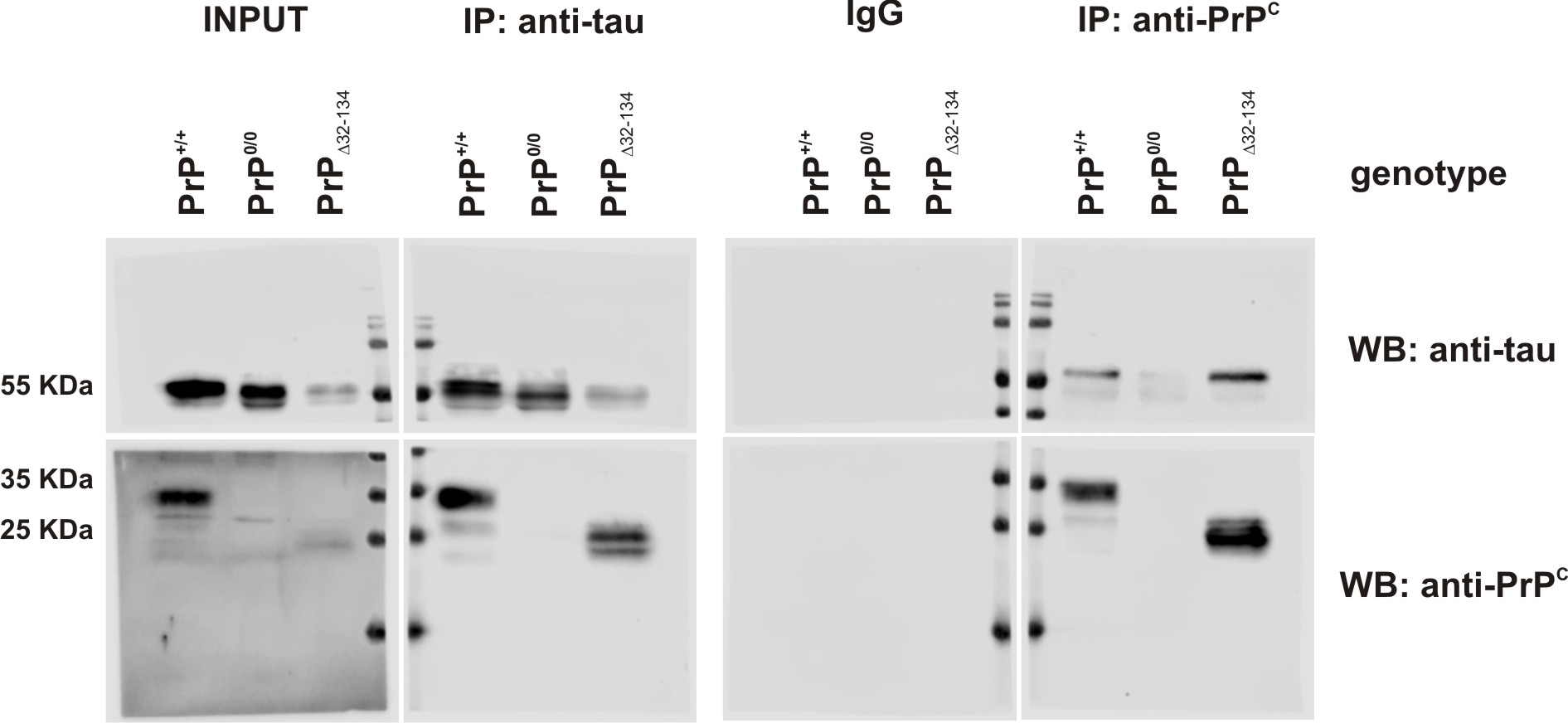

Supplement: Supplementary file 2 — Supplementary file2 (JPG 120 KB) [file 12035_2025_5163_MOESM2_ESM.jpg]
